# Factors Associated with Non-Attendance at Dental Preventive Care in Slovak High School Students

**DOI:** 10.3390/ijerph18031295

**Published:** 2021-02-01

**Authors:** Martin Samohyl, Jana Babjakova, Diana Vondrova, Jana Jurkovicova, Juraj Stofko, Branislav Kollar, Katarina Hirosova, Alexandra Filova, Lubica Argalasova

**Affiliations:** 1 Institute of Hygiene, Faculty of Medicine, Comenius University in Bratislava, Spitalska 24, 813 72 Bratislava, Slovakia; martin.samohyl@fmed.uniba.sk (M.S.); jana.babjakova@fmed.uniba.sk (J.B.); diana.vondrova@fmed.uniba.sk (D.V.); jana.jurkovicova@fmed.uniba.sk (J.J.); katarina.hirosova@fmed.uniba.sk (K.H.); alexandra.filova@fmed.uniba.sk (A.F.); 2 Institute of Physiotherapy, Balneology and Medical Rehabilitation, The University of St. Cyril and Methodius in Trnava, Námestie Jozefa Herdu 577/2, 917 01 Trnava, Slovakia; juraj.stofko@gmail.com; 3 Department of Neurology, Faculty of Medicine, Comenius University in Bratislava, University Hospital Bratislava, Mickiewiczova 13, 813 69 Bratislava, Slovakia; b.kollar@pobox.sk

**Keywords:** dental preventive care, adolescents, young adults, risk factors

## Abstract

This study aimed to determine the factors associated with the avoidance of dental preventive care in high school students and their parents in the framework of The Youth and Parents Risk Factor Behavior Survey in Slovakia, the ongoing cross-sectional school-based survey of students and their parents or legal representatives. The data were collected using two separate standardized questionnaires: (i) the questionnaire for students (*n* = 515) and (ii) the questionnaire for parents (*n* = 681). The study group included 57 high school students (54.4% males) who did not visit the dentist for preventive care in the previous year. The control group included 458 students (35.8% males) who visited a dentist for preventive care at least once in the previous year. A significantly higher number of males (54.4%), older adolescents, and young adults (21.8%; 20.0%) were not visiting dental preventive care regularly. Incomplete family (56.1%), stressful situations at home (17.5%), and feeling unwell were the factors contributing to the avoidance of dental preventive care. More than 34.5% of adolescents and young adults were not visiting either dental preventive care or pediatric preventive care (adjusted odds ratio (AOR) = 5.14; 95% confidence interval (CI) = 2.40, 10.99). Children of divorced mothers and mothers with household income lower than EUR 900 had significantly higher dental care avoidance in bivariate analysis. A significantly higher percentage of fathers from the exposed group were not visiting dental preventive care regularly (47.8%, *p* < 0.05). The results of the study can be used as an educational intervention step focusing on the parental influence on adolescent and young adults’ behavior and as a challenge for the improvement of dental preventive care in older adolescents and young adults.

## 1. Introduction

Despite being largely preventable, oral diseases and inequalities constitute a significant public health problem in the prevalence of the major chronic diseases of the 21st century. Oral health is an integral part of general health and should not be considered in isolation, as many risk factors of poor oral health [1] are the risk factors for obesity, heart disease, stroke, cancer, etc. [2]. 

Dental preventive care aims at reducing tooth decay incidence [3,4] and searching for dental disorders. The education of parents plays one of the key roles in prevention [5]. The most common chronic oral disease is dental caries in children, with a negative impact on health [6] and quality of life as a result of pain [7] and life-threatening infections [8]. Among oral diseases, which can be detected at dental preventive care, the most common are oral cancer, periodontal disease, trauma from injuries, oral infectious diseases, and hereditary lesions [9]. 

Adolescence is the life phase in which future patterns of maturity are created [10]. The processes of adolescence must be understood in the context of social influences and psychological development [11], and these processes can take up to about 25 years [12]. Young adults, as compared with other age groups, have the highest rate of death and injury from motor vehicles, homicides, mental health problems, sexually transmitted infections, and substance abuse. Most of the leading causes of illness and death among young adults are preventable [10]. Oral diseases are highly preventable and it is important to control the risk factors for oral diseases. Dental decay is perfectly preventable, but it is one of the most common chronic diseases [1]. 

The average number of teeth affected by dental caries has been declining from 1990 to 2015 in Europe (3.0 vs. 1.8). It was found that an average of 0.5–3.5 teeth are affected by dental caries in 12-year-old European children. In the European Region, 20–90% of 6-year-old children have at least one tooth decay. The preventive efforts of public health dentists in Slovenia under WHO guidance resulted in a significant reduction in the total caries index (DMFT) of 12-year-old children by 70%, from 5.1 in the year 1987 to 1.5 in the year 2017. The situation also improved in the Czech Republic and Slovakia [13].

According to Eurobarometer, the respondents the most likely to have visited a dentist during the past twelve months tend to be inhabitants of European Union countries: the Netherlands (83%), Denmark (78%), Germany, and Luxembourg (77%), followed by Slovakia (73%) and Sweden (71%). In some of these countries, it is compulsory for inhabitants to go to their dentist once a year or even every six months to continue to benefit from a medical insurance cover for their teeth [14]. 

In Slovakia, children under the age of 18 are allowed to visit a dentist twice a year for a preventive check-up without paying a fee. According to the latest available data in Slovakia, the number of persons with preventive examination per one inhabitant was 0.58 in the age group of 6–14-year-old inhabitants, 0.63 in the age group of 15–18-year-old adolescents, and 0.48 in the age group of 19+ year-old young adults The need for treatment was highest (69%) in the group of 19+ year-old young adults [15]. 

In the scientific studies accessible in the PubMed database for the period of the last five years, family income, female gender, dissatisfaction with oral health, and the attitude of parents were the most important factors related to the attendance at dental preventive check-ups in adolescents and young adults [16,17,18,19,20]. The recently published study in Slovakia provides representative findings on oral health related behaviours (OHRBs) in Slovakia and shows important associations of socioeconomic factors related to adolescents’ oral health issues in the age range of 10–16 years [21].

Our study is one of the most recent studies determining and analyzing the sociodemographic and socioeconomic factors and parental patterns associated with the avoidance of dental preventive care in the sample of adolescents and young adults (15–22 years old) in Bratislava, the capital of Slovakia, in the framework of The Youth and Parents Risk Factor Behavior Survey, an ongoing cross-sectional school-based survey of students and their parents or legal representatives.

The present study aimed to determine the factors associated with participation and avoidance of dental preventive care in the selected sample of high school students—adolescents and young adults, their parents, or legal representatives in the framework of an ongoing cross-sectional school-based survey. Concerning previous findings in scientific literature, we hypothesized that male gender, older age, socioeconomic status of the family, and parental pattern of dental preventive care avoidance could negatively impact the approach to dental preventive care in adolescents and young adults.

## 2. Materials and Methods 

The Youth and Parents Risk Factor Behavior Survey in Slovakia was initiated during the years 2015/2016 in Bratislava, the Slovak capital, as a model region [22,23]. It originates from the Behavioral Risk Factor Surveillance System (BRFSS) and The Youth Risk Behavior Surveillance System (YRBSS), originally designed by CDC, Atlanta, USA [22]. The BRFSS was a random telephone survey of US state residents aged 18 and older with the primary focus on such behaviors that include sedentary behavior, physical activity, nutrition, safety (e.g., the use of seatbelts and helmets), using tobacco and alcohol, getting preventive medical care, etc. [22]. The YRBSS was developed in 1990, monitoring six categories of priority health-risk behaviors among youth and young adults (aged 15–19 years) in public and private schools in the USA [3]. 

The data were collected using two separate standardized questionnaires: (i) the questionnaire for adolescents and (ii) the questionnaire for parents. They included questions on a residence, family, school, health and safety, habits and behavior, nutrition, body weight and height, lifestyle, and physical activity of adolescents and young adults and their parents. The special emphasis has been paid to the demographic (gender, age, residence, educational level) and behavioral (home and school stress, health status) characteristics of dental preventive care attendance among adolescents and young adults [24,25]. A pilot validation of the Slovak version of the questionnaire was performed on 20 respondents and then the questions were finalized.

The study was approved by the Ethics Committee of the Faculty of Medicine Comenius University and University Hospital on 25 July 2017 with the number 87/2016.

**Figure 1:**
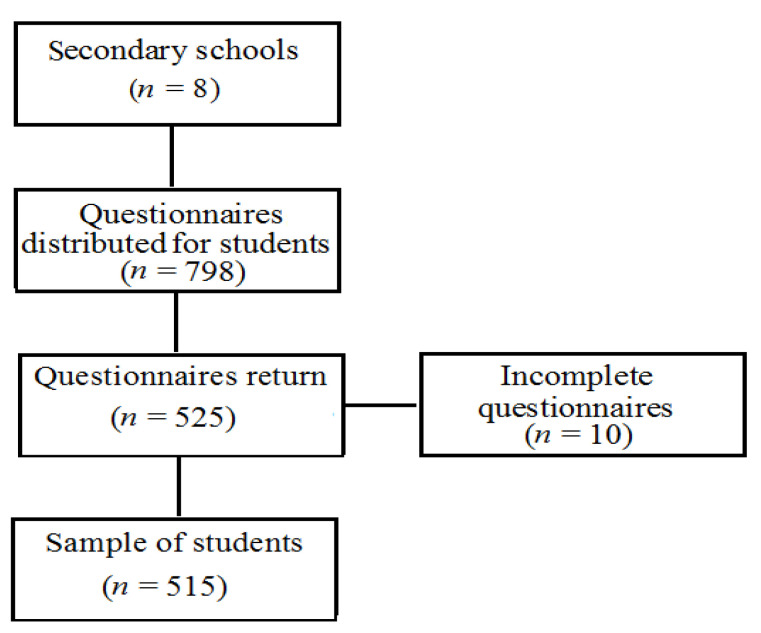
Scheme of the students’ sample (*n* = 515).

There were 2384 questionnaires distributed in total (798 for students and their 1586 parents), and the response rates were 64.0% and 66.1% respectively. Adolescents and young adults filled in questionnaires with the help of the trained personnel. Separate questionnaires were sent home to parents or legal representatives. Finally, the sample involved 515 adolescents and young adults aged 15−22 years old; 37.9% were males from 8 randomly selected secondary schools (two secondary grammar schools (40%); three vocational schools (30.4%) (hairdresser, make artists, masons, transportation); one school of art (3.2%); business academy (5.3%); and a nursing school (21.1%)) from a total of 101 secondary vocational and grammar schools and 22,723 students in Bratislava on the 1st January 2016 (Table 1, Figure 1).

In the present study, special attention was paid to the factors associated with the avoidance of dental preventive care in adolescents and young adults and their parents or legal representatives The study group included 57 high school students (mean age 17.2 ± 1.5 years; 54.4% males) who did not visit the dentist for preventive care in the previous year. The control group included 458 students (mean age 16.6 ± 1.3 years; 35.8% males) who visited a dentist for preventive care at least once in the previous year. The different number of adolescents (*n* = 515), their mothers (*n* = 390), and fathers (*n* = 291) in the study and control groups was due to the different response rate of the Questionnaire for Parents (mothers 75.7%, fathers 56.5%) and incompleteness of the family (28.7%) (Table 1).

### Data Analysis 

The data were analyzed using the Statistical Package for Social Science (SPSS) version 25 (International Business Machines Corp., New Orchard Road, Armonk, New York, USA). Descriptive statistics was used to obtain means, standard deviations, and proportions. The normality of the data distribution was verified by Shapiro Wilk Test. Associations between continuous variables (age) in the study group and the control group were analyzed by a two-sample t-test. Relationships between categorical data (gender, age groups, type of school, presence of stress at school and at home, residence, completeness of the family, feeling healthy, and pediatric preventive care visits) in both groups were evaluated by the chi-square test and Fisher exact test. The Fisher exact test was used in 2 × 2 tables when assumptions for the chi-square test did not hold. The statistically significant level was determined at *p* values of < 0.05. 

Multivariate analysis (multiple logistic regression) was performed to identify factors independently associated with non-attendance at dental preventive care in adolescents in the family, in mothers and fathers separately, mutually adjusted for all the variables (age, gender, family completeness, dental preventive care) using adjusted odds ratios and 95% confidence intervals. There were missing data in some variable categories. Missing data occurred because of no response or incompleteness of the family. There was no significant difference among occurrence of missing data in the exposed and control groups (χ^2^ = 0.649; *p* = 0.420). We considered them as random missing and not affecting the reported associations. Missing data were not included in the analysis.

## 3. Results

The demographic and socioeconomic characteristics of the sample of students are presented in Table 1. The highest number of students was in the age group of 16−17-year-olds (30.5%), mostly of Slovak nationality (91.1%). More than 10 percent of students were more than 19 years old. The majority of students attended grammar school (40.5%), had at least one sibling (82.5%), and lived in urban areas (59.4%). The complete family was observed in 71.3% of students. The majority of parents had completed secondary educational level (fathers: 64.1%; mothers: 62.5%). More than 91% of mothers and fathers were employed. 

Males (from the study group, *n* = 57) avoided dental preventive care more frequently than females (54.4% vs. 45.6%, *p* < 0.01). A significantly higher number of older students (18–19 years old, 19+ years old) (21.8, 20.0%) were not visiting dental preventive care regularly. Incomplete family (56.1%), stressful situations at home (17.5%), and feeling unwell were identified as the factors contributing to the avoidance of dental preventive care. More than 34.5% of adolescents and young adults were visiting neither dental preventive care nor pediatric preventive care (in Slovakia, pediatricians can have in their care children and youth up to 28 years of age) (Table 2). 

The influence of the mother on non-attendance at dental preventive care in the sample of adolescents and young adults has been demonstrated in bivariate analysis (Table 3). The social factors such as family income (lower than EUR 900) and incompleteness of the family (divorced and single mothers) were significantly associated with higher dental care avoidance in their children. It was found that in the study group there were significantly higher percentages of mothers who were not visiting preventive medical check-ups (32.4%) and dental care themselves (26.1%) (Table 3.)

The avoidance of preventive care (47.8%, *p* < 0.05) was the only variable representing the influence of the father on non-attendance at dental preventive care of students in bivariate analysis. 

Selected variables (male gender, the incompleteness of the family, pediatric preventive care attendance) were statistically significant among factors independently associated with non-attendance of students at dental preventive care in multivariate analysis (Table 4). 

Non-attendance at dental preventive care of the mothers was statistically significant (adjusted odds ratio (AOR) = 4.19; 95% confidence interval (CI) = 1.50, 11.71; *p* < 0.01) among variables independently associated with non-attendance at dental preventive care in adolescents and young adults in multivariate analysis (Table 4).

Single, divorced or widowed fathers (AOR = 3.73; 95%CI = 1.21, 11.54; *p* < *0*.05) and their non-attendance at dental preventive care (AOR = 3.41; 95%CI = 1.24, 9.32; *p* < 0.05) were statistically significant among variables independently associated with non-attendance at dental preventive care in adolescents and young adults in multivariate analyses (Table 4).

## 4. Discussion

This cross-sectional study identified several sociodemographic and socioeconomic factors associated with non-attendance at dental preventive care in the sample of adolescents and young adults and confirmed the assumptions hypothesized at the beginning of the study.

In the utilization of healthcare services, gender disparities are often present. In the cross-sectional study of Fonseca et al. [19] a significantly higher proportion of female adolescents (86.4%) as compared with males (82.9%) reported visiting the dentist in the past two years (*p* = 0.003). In this study, the percentage of adolescents who did not visit a dentist for more than two years was 15.1%, and it was significantly associated not only with gender, but also with lower family income, bad dental and periodontal health, and access to dental services and health care systems. In our study, males avoided dental preventive care more frequently than females (54.4% vs. 45.6%; *p*< 0.01). 

The American study on a large sample revealed that male gender, race, and lack of insurance were associated with a lack of annual dental visits [16]. In another American study by Ronis et al. [17], in a smaller number of face-to-face interviews socioeconomic status, race, and sex were important in planning regular dental visits. The study of Shaban et al. [18] confirmed a significant relationship between deprivation, family social class, and irregular dental visits of children. From Central and Eastern Europe, the Polish study showed that female gender and residence in a large city have a positive effect on visiting dental offices. The most frequent motivation for visiting a dental office was to receive conservative treatment, while the least common reasons were prophylaxis and tooth injuries [20]. The study on the large and nationally representative sample based on internationally elaborated study protocol of the Health Behaviour in School-aged Children study (HBSC) has been recently published in Slovakia [21]. The study provided representative findings on oral health related behaviours (OHRBs) in Slovakia and has shown important associations of socioeconomic factors related to adolescents’ oral health issues in the age range of 10–16 years. Besides preventive dental check-ups, they checked self-reported dental hygiene, toothbrush changing, and gum bleeding. Attending dental preventive check-ups was associated with female gender, lower age, higher family affluence, higher educational level, and employment of parents [21]. In the study by Aalsma et al. [26] analyzing parents’ and adolescents’ doubts about preventive care, a significant relationship between adolescents who discussed health with their parents and visiting the special medical provider on regular basis was found. One of the main barriers of not seeking preventive care in adolescents was their parents who avoided dental preventive medical visits. This was also confirmed in our study among mothers (AOR = 4.19; 95% CI = 1.50, 11.71) and fathers (OR = 3.41; 95% CI = 1.24, 9.32) in bivariate and multivariate analysis. Mothers and fathers who do not attend preventive dental check-ups regularly are more likely not to attend general preventive check-ups paid by health insurance. Non-attendance at dental preventive care among mothers and fathers remained significant among variables independently associated with non-attendance at dental preventive care in adolescents and young adults in multivariate analyses.

This finding can be partly explained by the parental behavior model where adolescents often observe the behavior of their parents. Discussion with parents about medical or dental care importance has a great impact on attendance at preventive check-ups. 

The age of adolescents and young adults has an effect on non-attendance at dental preventive care in bivariate analysis. A significantly higher number of older students (18–19 years old, 19+ years old) (21.8, 20.0%) were not visiting dental preventive care regularly. Multiple logistic regression (after adjustment for all the other variables) does not show a significant effect of age. 

In our study, 67.9% of students felt healthy and were not visiting general preventive care. Adolescents and young adults are thought to be healthy, but a lot of them have some health problems already [27]. Ensuring adequate health care in an adolescent and early adult age can be challenging compared with older adults and children, due to their rapid emotional, physical, and intellectual development [28].

Attendance at dental services also depends on socio-economic and individual characteristics. A significant impact on avoidance of dental preventive care had students with incomplete family and stressful situations at home. Adolescents with an incomplete family often feel a lack of social support, which can cause negative health behavior. Inadequate social support was associated with less frequent use of dental services [29]. Stress at home, as the individual variable responsible for dental care avoidance, can be influenced by socio-economic variables of mothers and fathers, such as divorced marital status and low household income. The divorced marital status has a great impact on an adolescent’s emotional well-being and household income [30].

Socioeconomic factors are very important as risk factors contributing to oral health. Students from bad socioeconomic conditions should be targeted primarily and more frequent dental check-ups should be asked for because there is a higher potential risk of dental caries due to bad dental hygiene. It is crucial to stress the importance of preventive dental check-ups in children, adolescents, and young adults at least twice a year, and also those over the age of 18 years. 

The improvement of the well-being and oral health of children, adolescents, and young adults can be achieved with the help of periodical and continuous education of students, parents, and teachers. 

The strength of our study is the fact that this is one of the few studies determining and analyzing the sociodemographic and socioeconomic factors and parental patterns associated with the avoidance of dental preventive care in the sample of adolescents and young adults in Slovakia in the framework of the international project the Youth and Parents Behavioral Survey (YABS). This is a comprehensive study based on a combination of two validated studies where parents are directly involved in the study. This causes challenges for the analysis and future prevention, intervention, and promotion of oral health in adolescents and young adults.

Nevertheless, this study has its limitations The first limitation of this study is the cross-sectional hypothesis-generating design that measures the outcome and the exposure in the study participants at the same time, and it is difficult to derive the causal relationship. The second limitation is the small sample size of the study group of adolescents and young adults reporting non-attending preventive dental check-ups in the previous year. However, there was not a big difference between groups in older adolescents and young adults. Those age groups that have stopped fulfilling the criteria for two preventive check-ups covered by health insurance (up to 18 years of age) are at special risk. This finding represents a challenge for future prevention and intervention. The third limitation is that the sample is representative only of adolescents and young adults attending high schools in the city of Bratislava. Students not attending high school due to disabilities and without access to these resources were excluded. The fourth limitation is that this is only the questionnaire survey, and the self-reported data do not allow us to get an actual picture of the previous dental experience and dental status of the respondents. The fifth limitation is the missing data. Missing data occurred because of no response or incompleteness of the family. There was no significant difference among occurrence of missing data in the exposed and control groups, and we considered them as random missing and not affecting the reported associations. 

## 5. Conclusions

Our study revealed several sociodemographic (male gender, older adolescent and young adult age), socioeconomic (incomplete family, low family income), and behavioral (stress at school, at home, subjective healthy feeling) factors which have a significant impact on the avoidance of dental preventive care in adolescents and young adults. 

A parental pattern in dental preventive care avoidance in parents also had a significant impact on non-attendance at dental preventive care in adolescents. 

In our preventive strategy, we should focus on those significant risk factors identified in the study in a targeted campaign, especially on family and parental patterns. Parents, students, and teachers should be aware that oral health is an integral part of general health and it impacts the overall quality of life. It affects also society and health systems through the associated health costs. The prevention of caries is important in children, adolescents, and young adults because oral diseases remain among the most important health burdens. We recommend the introduction of free preventive dental check-ups twice a year also for older adolescents and young adults over the age of 18 years. Within the consequences of health policy, we will contribute to the increased attendance of adolescents and young adults at preventive dental care, and thus to the reduction in the incidence of dental caries and also health care expenditures. 

## Figures and Tables

**Table 1:** Characteristics of the students´sample (*n* = 515) ^†.^

Variables		N (%)
Gender	Male	195 (37.9)
	Female	320 (62.1)
Age groups (yrs)	15–16	106 (20.6)
	16–17	157 (30.5)
	17–18	119 (23.1)
	18–19	79 (15.3)
	19+	59 (10.5)
Nationality *	Slovak	469 (91.1)
	Other	46 (8.9)
Type of school	Grammar	209 (40.5)
	Vocational	126 (24.3)
	Secondary	182 (35.2)
Siblings	Yes	425 (82.5)
	No	90 (17.5)
Residence	Urban	306 (59.4)
	Rural	209 (40.6)
Family	Complete	363 (71.3)
	Incomplete	146 (28.7)
Father’s education ^†^	Primary	8 (2.8)
	Secondary	186 (64.1)
	University degree	96 (33.1)
Mother’s education ^†^	Primary	9 (2.3)
	Secondary	243 (62.5)
	University degree	137 (35.2)
Employment status father ^†^	Employed	454 (93.8)
	Unemployed	30 (6.2)
Employment status mother ^†^	Employed	470 (91.3)
	Unemployed	45 (8.7)

^†^ There were missing data in some variable categories. Missing data occurred because of no response or incompleteness of the family; * other nationalities were Czech, Ukrainian, and Serbian.

**Table 2:** Characteristics of selected variables associated with non-attendance at dental preventive care in the sample of adolescents (*n* = 515) ^†.^

Variables		Study Group ^1^	Control Group ^2^	*p*-Value
		N (%) ^†^ (*n* = 57)	N (%) ^†^ (*n* = 458)	
Gender	Male	31 (54.4)	164 (35.8)	0.010
	Female	26 (45.6)	294 (64.2)	
Age groups (yrs)	x ± SD	17.2 ± 1.5	16.6 ± 1.3	0.004
	15–16	5 (9.1)	100 (22.1)	0.025
	16–17	16 (29.1)	140 (30.9)	
	17–18	11 (20)	107 (23.6)	0.018 ^4^
	18–19	12 (21.8)	65 (14.3)	
	19+	11 (20)	41 (9.1)	0.011
Type of school	Grammar	18 (31.6)	191 (41.7)	0.309
	Vocational	18 (31.6)	106 (23.1)	
	Secondary	21 (36.8)	161 (35.2)	
Residence	Urban	35 (61.4)	271 (59.2)	0.747
	Rural	22 (38.6)	187 (40.8)	
Family	Complete	25 (43.9)	338 (74.8)	<0.001
	Incomplete	32 (56.1)	114 (25.2)	
Stress at home	Yes	10 (17.5)	29 (6.5)	0.037
	No	47 (82.5)	420 (93.5)	
Stress at school	Yes	31 (54.4)	223 (48.7)	0.422
	No	26 (45.6)	235 (51.3)	
Healthy feeling	Yes	38 (67.9)	397 (86.9)	0.005
	No	18 (32.1)	60 (13.1)	
Pediatric preventive care ^3^	Yes	36 (65.5)	421 (91.5)	<0.001
	No	19 (34.5)	39 (8.5)	

^1^ Adolescents who did not visit the dentist in the previous year; ^2^ adolescents who visited a dentist for preventive care at least once in the previous year; ^3^ at least once a year; ^†^ there were missing data in some variable categories; ^4^ statistical significance between study and control group in all age groups. Missing data occurred because of no response or the incompleteness of the family.

**Table 3:** Characteristics of selected variables in mothers associated with non-attendance at dental preventive care in the sample of adolescents (*n* = 390) ^†.^

Selected Variables in Mothers		Study Group ^1^	Control Group ^2^	*p*-Value
		N (%) ^†^ (*n* = 35)	N (%) ^†^ (*n* = 355)	
Age groups (yrs)	x ± SD	42.4 ± 4.4	43.6 ± 5.1	0.183
	30–50	33 (94.3)	321 (90.4)	0.371
	51–71	2 (5.7)	34 (9.6)	
Mother’s educational level	Primary school	2 (5.7)	7 (2.0)	0.259
	High school	23 (65.7)	220 (62.1)	
	University	10 (28.6)	127 (35.9)	
Marital status	Single	4 (11.4)	25 (7.0)	0.034
	Married	16 (45.7)	263 (74.1)	
	Divorced	13 (37.1)	58 (16.3)	
	Widow	2 (5.8)	9 (2.6)	
Residence	Urban	22 (62.9)	217 (61.1)	0.843
	Rural	13 (37.1)	138 (38.9)	
Employment	Yes	30 (88.2)	317 (89.8)	0.789
	No	4 (11.8)	36 (10.2)	
Household income (EUR)	≤900	19 (55.9)	115 (35.6)	0.031
	>900	15 (44.1)	208 (64.4)	
Stress at work	Yes	24 (75.0)	244 (73.5)	0.854
	No	8 (25.0)	88 (26.5)	
Healthy feeling	Yes	29 (82.9)	291 (84.3)	0.826
	No	6 (17.1)	54 (15.7)	
Preventive care ^3^	Yes	23 (67.6)	309 (88.5)	0.017
	No	11 (32.4)	40 (11.5)	
Dental preventive care	Yes	17 (73.9)	293 (95.1)	0.034
	No	6 (26.1)	15 (4.9)	

^1^ Adolescents who did not visit the dentist in the previous year; ^2^ adolescents who visited a dentist for preventive care at least once in the previous year; ^3^ preventive care in at least one physician (gynecologist, urologist, gastroenterologist, GP) at least once a year; ^†^ there were missing data in some variable categories. Missing data occurred because of no response or the incompleteness of the family.

**Table 4:** Characteristics of selected variables associated with non-attendance at dental preventive care of students in students (*n* = 515), mothers (*n* = 390), and fathers (*n* = 291) multivariate analysis ^†^.

	Variables		AOR	95%CI
**Selected variables in students**	Gender	Female	1	-
		Male	2.11	1.09, 4.03 *
	Age groups (yrs)	15–18	1	-
		19+	1.19	0.51, 2.78
	Type of school	Grammar	1	-
		Secondary/Vocational	1.32	0.65, 2.70
	Residence	Rural	1	-
		Urban	0.77	0.08, 7.64
	Family	Complete	1	-
		Incomplete	3.26	1.68, 6.30 ***
	Stress at home	No	1	-
		Yes	1.71	0.62, 4.68
	Stress at school	No	1	-
		Yes	1.18	0.61, 2.28
	Healthy feeling	Yes	1	-
		No	1.13	0.49, 2.60
	Pediatric preventive care ^1^	Yes	1	-
		No	5.14	2.40, 10.99 ***
**Selected variables in mothers**	Age group (yrs)	30–50	1	-
		51–71	0.40	0.05, 3.21
	Mother’s educational level	High school diploma/University	1	-
		Primary/High school without a diploma	1.20	0.40, 3.62
	Marital status	Married/in relationship	1	-
		Single/divorced/widowed	2.20	0.91, 5.32
	Residence	Rural	1	-
		Urban	1.10	0.45, 2.66
	Employment	Yes	1	-
		No	0.42	0.04, 4.41
	Household income (EUR)	>900	1	-
		≤ 900	1.51	0.63, 3.63
	Stress at work	No	1	-
		Yes	0.97	0.67, 2.54
	Healthy feeling	Yes	1	-
		No	0.86	0.25, 2.98
	Preventive care ^2^	Yes	1	-
		No	1.95	0.69, 5.46
	Dental preventive care	Yes	1	-
		No	4.19	1.50, 11.71 **
**Selected variables in fathers**	Age group (yrs)	32–44	1	-
		45–76	0.50	0.19, 1.27
	Father’s educational level	High school diploma/University	1	-
		Primary/High school without a diploma	1.50	0.56, 4.02
	Marital status	Married/in relationship	1	-
		Single/divorced/widowed	3.73	1.21, 11.54 *
	Residence	Rural	1	-
		Urban	0.91	0.34, 2.46
	Employment	Yes	1	-
		No	-	-
	Household income (EUR)	>900	1	-
		≤900	0.48	0.14, 1.57
	Stress at work	No	1	-
		Yes	0.47	0.18, 1.24
	Healthy feeling	Yes	1	-
		No	1.98	0.56, 6.98
	Preventive care ^3^	Yes	1	-
		No	1.23	0.43, 3.50
	Dental preventive care	Yes	1	-
		No	3.41	1.24, 9.32 *

AOR—adjusted odds ratio for all mutually adjusted variables; CI—confidence interval; ^1^ at least once a year; ^2^ preventive care from at least one physician (gynecologist, urologist, gastroenterologist, GP) at least once a year; ^3^ preventive care from at least one physician (urologist, gastroenterologist, GP) at least once a year; * *p* < 0.05; ** *p* < 0.01; *** *p* < 0.001; ^†^ there were missing data in some variable categories. Missing data occurred because of no response or incompleteness of the family. After exclusion of missing data, the multiple logistic regression model included 479 adolescents, 325 mothers, and 265 fathers.

## Data Availability

The data presented in this study are available on request from the corresponding author.
